# Plasminogen Activator Inhibitor-1 in depression: Results from Animal and Clinical Studies

**DOI:** 10.1038/srep30464

**Published:** 2016-07-26

**Authors:** Haitang Jiang, Xiaoli Li, Suzhen Chen, Na Lu, Yingying Yue, Jinfeng Liang, Zhijun Zhang, Yonggui Yuan

**Affiliations:** 1 Department of Psychosomatics and Psychiatry, ZhongDa Hospital, Medical School of Southeast University, Nanjing 210009, P. R. China; 2 Institute of Psychosomatics, Medical School of Southeast University, Nanjing 210009, P. R. China; 3 Department of Neurology, ZhongDa Hospital, Neuropsychiatric Institute, Medical School of Southeast University, Nanjing 210009, P. R. China

## Abstract

Evidence suggests that plasminogen activator inhibitor-1 (PAI-1) is a stress-related factor, and serum PAI-1 levels are increased in patients with major depressive disorders (MDD). Herein, we analysed PAI-1 protein levels in the brain, cerebrospinal fluid (CSF) and serum of rodents exposed to chronic unpredictable mild stress or treated with escitalopram. In addition, we examined PAI-1 concentrations in serum obtained from 17 drug-free depressed patients before and after escitalopram treatment. We found that PAI-1 expression was increased in area 1 of the cingulate cortex and prelimbic cortex of the medial prefrontal cortex as well as in the hippocampal cornu ammonis 3 and dentate gyrus in stressed rats. A downregulation of PAI-1 following chronic escitalopram treatment was also found. PAI-1 levels were higher in the CSF and serum in stressed rats than in controls, although the difference did not reach statistical significance in the serum. Escitalopram treatment significantly decreased PAI-1 levels in the serum, but not in the CSF. MDD patients had significantly greater serum PAI-1 concentrations than controls. Our results suggest that PAI-1 is implicated in the pathophysiology of depression.

Major depressive disorder (MDD) is one of the most common psychiatric disorders and affects more than 350 million people in the world^1^. As a major disabling medical condition, MDD is predicted to be the foremost contributor to the worldwide disease burden by 2030^2^.

However, the underlying mechanisms remain unknown, and treatments are inadequate. Recent animal and human studies have suggested that the tissue-type plasminogen activator (tPA)/plasminogen system might play a vital role in MDD pathophysiology^3^,^4^. tPA is a major plasminogen activator that converts inactive plasminogen into an active protease, plasmin, which efficiently breaks down fibrin clots, helping to restore vascular patency. Plasminogen activator inhibitor-1 (PAI-1) is the most important inhibitor of tPA^5^. Recent evidence suggests that low tPA levels related to increased levels of its inhibitor PAI-1 are associated with stress response and depression^3^,^4^,^5^.

The mechanism by which the deletion of tPA relates to depression-like and anxiety-like behaviours^6^, seems to involve brain derived neurotrophic factor (BDNF). BDNF precursor (proBDNF) is proteolytically cleaved into mature BDNF by tPA and by plasmin, tPA end product^7^. Whereas proBDNF favors neuronal apoptosis and long-term depression, mature BDNF has anti-apoptotic properties and favors long-term potentiation^8^. Cleavage of proBDNF by tPA/plasmin is also essential for long-term hippocampal plasticity^7^. Physical exercises that have antidepressant effects also increase the tPA and mature BDNF levels^4^. The inability to convert proBDNF into mature BDNF is associated with MDD pathogenesis. As expected, mature BDNF is implicated in the mechanism of action of antidepressants^9^.

PAI-1, the tPA inhibitor, might be involved in the pathogenesis of MDD through inhibition of the tPA/plasminogen system and proBDNF cleavage. It has been demonstrated that PAI-1 protein expression was increased in the prefrontal cortex and hippocampus of rats showing depression-like behaviours^10^. Depressed patients have higher serum concentrations of PAI-1^11^,^12^. Tsai and colleagues^13^ also showed that two variants (rs2227684-G and rs7242-T alleles) of the PAI-1 gene (SERPINE1) are more frequent in MDD patients than in controls. However, PAI-1 levels in the cerebrospinal fluid (CSF) and serum of rats with depression-like behaviours and the behaviour of PAI-1 during antidepressant treatment in subregions of the prefrontal cortex and hippocampus remain to be defined. It is also unclear if MDD serum concentrations of PAI-1 are modulated by antidepressant treatment.

In this study, using chronic unpredictable mild stress (CUMS) as an animal model of depression, we investigated the main brain areas that contribute to depression and sought to understand the role of PAI-1 in depression and its behaviour in antidepressant treatment. We also determined PAI-1 levels in the CSF and serum during stress or escitalopram treatment in animals. We also aimed to assess if serum PAI-1 was altered in drug-free MDD patients, observe their putative changes during antidepressant treatment.

## Material and Methods

### Animals

**Figure 1:**
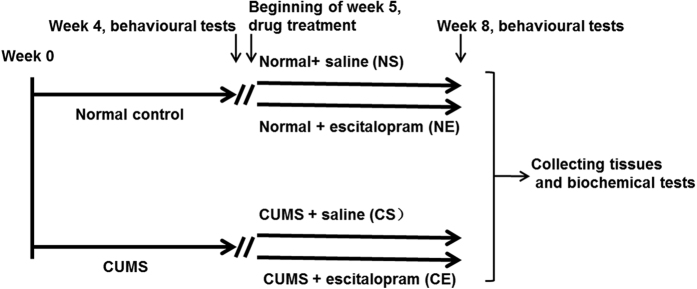
Chronic unpredictable mild stress procedure and drug treatment. After one week of acclimation, rats were randomly assigned to the following two groups: CUMS and normal. CUMS rats were exposed to chronic mild stress for four weeks and then subjected to three behavioural paradigms (SPT, FST, OFT tests). Only the rats that exhibited depressive-like behaviours in at least two of the three tests were chosen for the following experiments. At the beginning of week five, we randomly allocated the stressed rats to the following two groups: CUMS + saline (CS, n = 6/group) and CUMS + escitalopram (CE, n = 6/group). Meanwhile, the normal controls were also divided into the following two groups: normal+ saline (NS, n = 6/group) and normal + escitalopram (NE, n = 6/group). For a total of 4 weeks, the CS and CE groups were exposed to stressors and received saline or escitalopram treatment, and the NS and NE groups received saline or escitalopram treatment. Then, all of the rats were subjected to the three behavioural paradigms. Then, we collected the cerebrospinal fluid and serum and dissected brain regions for biochemical tests. CUMS: chronic unpredictable mild stress procedures; SPT: sucrose preference test; FST: forced swimming test; OFT: open-field test.

We used adult male 180–220 g Sprague-Dawley rats (Experimental Animal Center of Shanghai). These rats were maintained on a 12 h light-dark cycle in a temperature-controlled environment. The rats had free access to food and water except during experimental procedures. A figure was made to illustrate the process of the chronic unpredictable mild stress procedures, drug treatments and biochemical tests (Fig. 1). The animals were cared for in accordance with the approved guidelines of the National Institutes of Health for Animal Care. The experimental protocols were approved by the Jiangsu Animal Care and Use Committee at Southeast University (NO. 20140225006).

### Chronic unpredictable mild stress (CUMS) procedure and response to treatment

The procedural details of CUMS and the behavioural paradigms were described previously^14^. After one week of acclimation, the rats were randomly assigned to the following two groups: CUMS and control. The chronic stress lasted for eight weeks and included eight different stressors, which were applied randomly. These stressors comprised the following: food deprivation, water deprivation, continuous light period, grouped housing, soiled cage, tail clamping, cage tilting and cold water swimming^14^,^15^. After four weeks of exposure to chronic mild stress, the CUMS rats were subjected to the following three behavioural paradigms: sucrose preference test (SPT), forced swimming test (FST), and open-field test (OFT). Only the rats that exhibited depression-like behaviours in at least two of the three tests were chosen for the proceeding experiments. We randomly allocated the stressed rats to the following two groups: CUMS + saline (CS, n = 6/group) and CUMS + escitalopram (CE, n = 6/group). The normal controls were divided into the following two groups: normal + saline (NS, n = 6/group) and normal + escitalopram (NE, n = 6/group). Escitalopram oxalate (kindly provided by H. Lundbeck A/S. Copenhagen-Valby, Denmark) was freshly dissolved into a saline solution. Escitalopram was injected intraperitoneally daily into the NE and CE rats at a volume of 1 ml/kg body weight and at a dose of 10 mg/kg body weight from week five, which was after a total of 4 weeks of exposure to the stressors. The NS and CS groups received equivalent injections of the saline solution.

### Behaviour testing

The SPT, FST, OFT and body weight paradigms were performed three times as follows: at the beginning, middle, and the end of the experimental period. The final behavioural tests were carried out 48 hours after the last dose of escitalopram administration. A brief description of these tests is as follows: i) *Sucrose Preference Test*: The SPT was performed to assess anhedonia^16^,^17^. Animals were given free access to two bottles- one containing 1% sucrose solution and the other containing water. The bottle positions were changed after 12 hours. The test lasted for 24 hours. By weighing the bottles at the beginning and end of each test, the sucrose preference was calculated as follows: sucrose preference (%) = sucrose intake/(sucrose intake + water intake) × 100%^17^. ii) *Forced Swimming Test:* For the FST, we placed the rats individually into a cylindrical tank (30 cm diameter × 60 cm height; Shanghai Yishu Company, Shanghai, China) that was filled with water (25 °C, 30 cm deep). The test lasted for six minutes. We analysed the immobility only during the last five minutes. A video camera placed in front of the cylindrical tank recorded the rat behaviour. A rat was classified as showing behavioural immobility when it reduced the intensity of movement, only producing the necessary movements to maintain its head above water^18^. iii) *Open Field Test:* Spontaneous locomotor and rearing activity were measured using the OFT between 2 and 5 pm. Briefly, the test apparatus consisted of a wooden box (100 × 100 × 50 cm). We placed each rat in the centre of the open field and allowed it to move freely. The total distance and rearing times of each rat were scored over a 5-minute period and recorded by a video camera. The apparatus was cleaned with detergent to eliminate odours prior to each test.

### ELISA

After the CUMS procedures and behavioural tests were completed, rats from the above four groups were anesthetized with 10% chloral hydrate. We used a stereotaxic apparatus with ear bars to hold the anesthetized rats in place. We made a longitudinal incision (approximately 2 cm) beginning between the ears and ending caudally. We then directly inserted a microliter syringe needle (Gaoge, China) from the lateral posterior of the foramen magnum into the cisterna magna and withdrew CSF. We centrifuged the CSF at 2000 rpm at 4 °C for 20 min and stored it at −80 °C until use for further assay. After withdrawing the CSF, we quickly opened the rat, inserted a disposable venous blood lancet directly into the left ventricle of heart, and collected the blood into a plain tube. The blood was centrifuged at 2000 rpm at 4 °C for 20 min, and the supernatants (serum) were frozen at −80 °C. The active PAI-1 concentrations in the serum and CSF were measured using enzyme linked immune sorbent assay [ELISA, PAI-1 (SERPINE1): ab198509, abcam] according to the manufacturer’s instructions. Each sample was tested in duplicate. The minimum detectable dose was 0.08 ng/ml.

### Western blot

After collected the CSF and blood, we decapitated five rats from each group, rapidly removed their brains, and stored the brains at −80 °C until further use. We sectioned the frozen cerebrum at 200 μm thick using Cryostat Microtome (CM1950, Leica, Germany) and dissected area 1 of the cingulate cortex and prelimbic cortex from the medial prefrontal cortex as well as the cornu ammonis 1, cornu ammonis 3 and dentate gyrus from the hippocampus. Briefly, according to the sectional anatomy atlas of rat brains^19^, prelimbic cortex and area 1 of the cingulate cortex were removed from the Bregma 3.70 mm to Bregma 2.20 mm, and the dorsal hippocampus was removed from the Bregma −3.30 mm to Bregma −4.30 mm. The cornu ammonis 1, cornu ammonis 3 and dentate gyrus regions were removed using brain matrices and a stereoscopic anatomic microscope^14^. The protein concentration of each brain area homogenate was >6.2 μg/μl. The amount of homogenate protein loaded in each gel was 30 μg. Protein extracts of the tissues were separated on sodium dodecyl sulfate polyacrylamide gel electrophoresis (SDS-PAGE) gels. After electrophoresis, the proteins were transferred onto polyvinylidene fluoride membranes and blocked with 5% skim milk in Tris-buffered saline containing 0.05% Tween 20 (TBST). The membranes were incubated with primary antibodies for PAI-1 (1:200, Santa Cruz) and glyceraldehyde-3-phosphate dehydrogenase (GAPDH) (1:1000, Abcam) overnight. After the membranes were washed, they were incubated with goat anti-rabbit (1:5000, Thermo). The film signal was digitally scanned and then quantified using Quantity One software (Bio-Rad). We used GAPDH as the loading control, and the PAI-1 levels were normalized to the GAPDH concentrations.

### Human subjects

A total of 17 MDD inpatients (3 males and 14 females) undergoing a major depressive episode were recruited from the Department of Psychosomatics and Psychiatry Affiliated ZhongDa Hospital of Southeast University. The patients met the diagnostic criteria for MDD according to the Diagnostic and Statistical Manual of Mental Disorders, 4th Edition (DSM-IV)^20^. All of the patients had been drug free for at least 2 weeks before entering the study. We used the 17-item Hamilton Depression Rating Scale (HDRS)^21^ to assess the severity of the depression. The exclusion criteria for all patients were comorbid Axis I diagnosis, alcohol or substance abuse or dependence, or other neurological illnesses, including dementia or stroke. Treatment began after an initial assessment with HDRS. We collected blood samples on the day before drug treatment initiation. The patients were administered escitalopram at a mean dose of 12.35 mg/day. All of the patients were co-treated with benzodiazepines during this period. At the end of the eighth week, we again conducted an HDRS assessment and blood sample collection. The response to treatment was defined as a reduction of >50% of the HDRS score from baseline to week eight.

We also recruited 17 healthy controls (4 males and 13 females) from the general community. None of these controls had a history of DSM-IV Axis I or II disorder, neurological illness, alcohol or drug abuse and dependence. Serum samples were taken after being evaluated by HDRS. Their HDRS scores were all <7. After a full written and verbal explanation of the study, all subjects signed a written informed consent form. The Human Participants Ethics Committee of ZhongDa Hospital of Southeast University approved all procedures (NO. 2013ZDSYLL011.0), which adhered to the guidelines of the Declaration of Helsinki.

### Blood collection and ELISA

We collected venous blood samples in coagulant tubes between 6:30–8:00 am. The blood samples were centrifuged at 3000 rpm at 4 °C for 20 min, and the serum was stored at −80 °C until further assay. PAI-1 serum concentrations from each subject were measured by ELISA (PAI-1; DSE100; R&D Systems) on the same day to minimize assay variance. All experiments were performed in duplicate.

### Statistical analysis

For the animal study, we used GraphPad Prism 6.01 software to analyse the data, which were expressed as the mean ± standard error of mean. We considered the rat behaviour to be abnormal when the value was 2 standard deviations (SD) lower than that of the controls. Analysis of variance (ANOVA) was used to analyse the body weight change and behavioural tests at week 0 and week 4. Two-way ANOVA was applied for analysis of the data at week 8, followed by Turkey post-hoc test, including body weights, behavioural tests and PAI-1 concentrations in the brain tissue, CSF and serum. The body weight was treated as a covariate for the analysis of OFT and FST.

For human studies, we expressed the data as the mean ± SD and analysed the data with SPSS 17.0 software. Chi-square tests were performed on the categorical data of gender. We applied analysis of variance (ANOVA) to compare the age, education and body weight, HDRS scores and PAI-1 levels between the patients and controls. Then, we applied analysis of covariance to compare the PAI-1 levels between the above two groups by controlling for age, education and body weight. The analysis of a paired-samples t-test was used to compare the PAI-1 levels and HDRS scores in patients between the pre- and post-treatment. The relationships between the serum PAI-1 and clinical variables in drug-free patients were examined using Pearson’s correlation. The statistical threshold was set to *P* < 0.05.

## Results

### Effects of CUMS and escitalopram treatment on body weight and behaviour

#### Body weight and sucrose preference test

After four weeks of CUMS exposure, the body weight of stressed rats was decreased compared with controls (*P* < 0.001, n = 12/group). Chronic escitalopram treatment did not increase body weight in the CE group compared with the CS group (*P*_Turkey_ < 0.001, n = 6/group). Chronic escitalopram treatment also did not affect body weight in the unstressed or stressed groups (*P*_2-way ANOVA_ = 0.915, n = 6/group) (Table 1). The baseline sucrose preference in the CUMS group was not significantly different from the control group (*P* = 0.321, n = 12/group). Stress decreased the sucrose preference at the end of week four (*P* = 0.001, n = 12/group). Escitalopram treatment upregulated the sucrose preference ratio in the CS rats compared with the NS rats (*P*_Turkey_ < 0.001, n = 6/group), but not in the NE rats compared with the CS rats (*P*_Turkey_ = 0.849, n = 6/group) (Table 1).

#### Forced Swimming test

The immobility time of each group presented no significant difference before the beginning of the CUMS procedure (*P* = 0.838, n = 12/group). Four weeks of the CUMS procedure caused a significant increase in the immobility time (*P* < 0.001, n = 12/group), but chronic escitalopram treatment reversed this change (*P*_2-way ANOVA_ = 0.001, *P*_Turkey_ = 0.008, n = 6/group) (Table 1).

#### Open field test

Four weeks of CUMS procedure decreased the total distance in the OFT (*P* < 0.001), whereas escitalopram increased the total distance (*P*_2-way ANOVA_ < 0.001, *P*_Turkey_ = 0.001, n = 6/group). The rearing times were decreased at week four in the CUMS group compared with the normal control group (*P* < 0.001). Four weeks of chronic escitalopram treatment reversed this change (*P*_2-way ANOVA_ < 0.001, *P*_Turkey_ = 0.030, n = 6/group) (Table 1).

### PAI-1 in brain subregions, CSF and serum

#### PAI-1 in brain subregions

**Figure 2:**
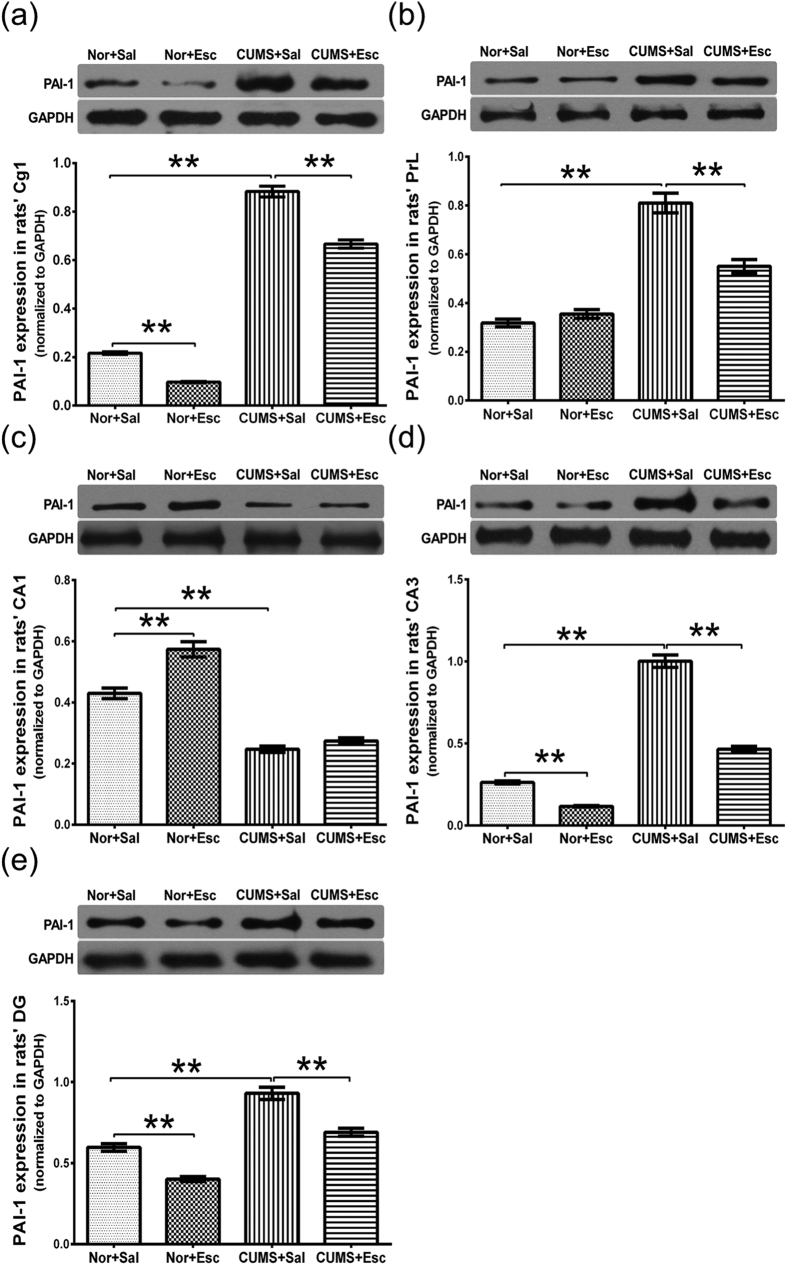
Protein Levels of PAI-1 in subregions of mPFC (**a,b**) and hippocampus (**c–e**). (**a,b**) CUMS increased PAI-1 levels in Cg1 and PrL of mPFC. This increase was decreased by escitalopram treatment. **(c)** There was a decrease in PAI-1 expression in the CA1 of the hippocampus in CUMS in saline treated subgroup rats, but this decrease was not reversed by escitalopram treatment. **(d**,**e)** Stress increased the expression of PAI −1 in both the CA3 and DG of the hippocampus. Escitalopram treatment reversed these changes in both of the subregions. The data are expressed as the mean ± standard error of mean (n = 5/group). The protein levels of PAI-1 were normalized to GAPDH. *P* values were obtained by post-hoc analysis, *P < 0.05 and **P < 0.01. mPFC: medial prefrontal cortex; CUMS: chronic unpredictable mild stress; Cg1: area 1 of the cingulate cortex; PrL: prelimbic cortex; CA1: cornu ammonis 1; CA3: cornu ammonis 3; DG: dentate gyrus; Nor: normal; Esc: escitalopram; GAPDH: glyceraldehyde-3-phosphate dehydrogenase.

Stress and escitalopram both significantly changed PAI-1 expression in the area 1 of the cingulate cortex (*P*_2-way ANOVA_ < 0.001 for each, n = 5/group). We observed an interaction effect of stress and escitalopram on the expression of PAI-1 (*P* = 0.004, supplementary Table S1). There was a significant increase of PAI-1 expression in the area 1 of the cingulate cortex of CS rats compared with NS rats (*P*_Turkey_ < 0.001), and this increase was reversed by escitalopram treatment in the CE rats compared with the CS rats (*P*_Turkey_ < 0.001; Table 2, Fig. 2a).

Stress and escitalopram also changed the concentration of PAI-1 in the prelimbic cortex (*P*_2-way ANOVA_ < 0.001 for each, n = 5/group). A significant interaction effect of stress and escitalopram was observed on the expression of PAI-1 (*P*_2-way ANOVA_ < 0.001, supplementary Table S1). CUMS increased the protein level of PAI-1 (*P*_Turkey_ < 0.001); however, escitalopram treatment decreased this level significantly (*P*_Turkey_ < 0.001; Table 2, Fig. 2b).

Stress and escitalopram both changed the PAI-1 expression in the cornu ammonis 1 region, (*P*_2-way ANOVA_ < 0.001 for each, n = 5/group). We also observed an interaction effect of stress and escitalopram (*P*_2-way ANOVA_ = 0.003, supplementary Table S1). PAI-1 expression was decreased in the cornu ammonis 1 region of the CS rats compared with the NS rats (*P*_Turkey_ < 0.001, Table 2); this decrease, however, was not reversed by escitalopram treatment in the CE rats compared with the CS rats (Table 2, Fig. 2c).

Stress increased the expression of PAI-1 in the cornu ammonis 3 and dentate gyrus regions (*P*_2-way ANOVA_ < 0.001 for each, n = 5/group, supplementary Table S1). Escitalopram treatment decreased the PAI-1 expression in both subregions of the hippocampus (*P*_2-way ANOVA_ < 0.001 for cornu ammonis 3 and dentate gyrus, n = 5/group, supplementary Table S1). The protein levels of PAI-1 were higher in the CS group in both the cornu ammonis 3 and dentate gyrus than in the NS group (all *P*_Turkey_ < 0.001). Escitalopram treatment decreased PAI-1 in the CE group compared with the CS group and NS group (*P*_Turkey_ < 0.001 for cornu ammonis 3 and dentate gyrus; Table 2, Fig. 2d,e).

#### Active PAI-1 in CSF and serum of rats

**Figure 3:**
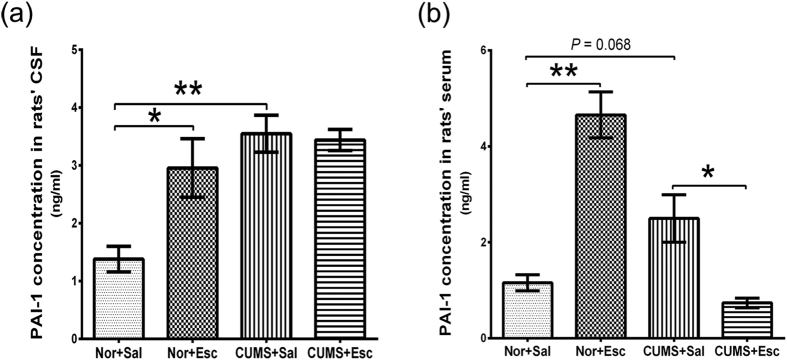
Active PAI-1 in rat cerebrospinal fluid (CSF) (**a**) and serum (**b**) of rats. **(a)** The active PAI-1 concentration (ng/ml) in CSF was increased by stress in the CS group, whereas escitalopram did not change the level in the CE group compared with the CS group. **(b)** There was a trend for increased active PAI-1 (ng/ml) in the serum of the CS group compared with the NS group, which decreased with escitalopram treatment. However, escitalopram treatment increased active PAI-1 expression in the NE group. The data are expressed as the means ± standard error of mean (n = 6/group). *P < 0.05 and **P < 0.01. CSF: cerebrospinal fluid. Nor: normal; Esc: escitalopram. CS: CUMS + saline; CE: CUMS + escitalopram; NS: normal + saline; NE: normal + escitalopram.

The active PAI-1 concentration in the CSF was significantly increased by stress in the CS group (*P*_Turkey_ < 0.001), whereas escitalopram did not change the expression of active PAI-1 in the CE group compared with the CS group (*P*_Turkey_ = 0.996). However, escitalopram increased the expression of PAI-1 in the NS group compared with the CS group (*P*_Turkey_ = 0.016) (Table 2, Fig. 3a). The interaction effect of stress and escitalopram was observed (*P*_2-way ANOVA_ = 0.020, supplementary Table S1). There was a trend for increased active PAI-1 in the serum of the CS group compared with the NS group (*P*_Turkey_ = 0.068), which was decreased by escitalopram treatment (*P*_Turkey_ = 0.012). However, escitalopram treatment increased the active PAI-1 expression in the NE group (*P*_Turkey_ < 0.001) (Table 2, Fig. 3b). A significant interaction effect of stress and escitalopram on serum levels of active PAI-1 was observed (*P*_2-way ANOVA_ < 0.001, supplementary Table S1).

### Measurement of PAI-1 concentration in the serum of MDD patients

#### Characteristics of MDD patients and controls

The demographic and neuropsychological characteristics of all of the subjects are presented in Table 3. No significant differences in gender, age, education and body weight were observed between MDD patients and healthy controls. The HDRS scores of the MDD patients were significantly decreased after 8 weeks of escitalopram treatment (*P* < 0.001).

#### PAI-1 in serum of MDD patients

**Figure 4:**
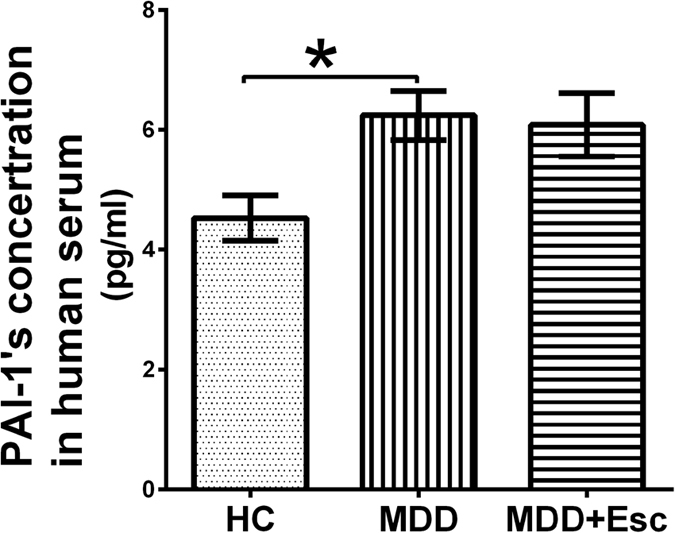
PAI-1 in serum of MDD patients. Serum PAI-1 concentrations (pg/ml) in MDD patients at baseline were significantly lower compared with controls, and this change was not significantly reversed by escitalopram treatment. The data are expressed as the mean ± standard deviation (n = 17/group). *P < 0.05. HC: healthy control; MDD: major depressive disorder; Esc: escitalopram.

The baseline serum PAI-1 concentrations in the MDD patients were significantly higher compared with controls (*P* = 0.004). The difference was still significant (*P* = 0.049) after controlling for the effects of age, education and body weight. However, escitalopram treatment did not change the PAI-1 levels (*P* = 0.705) (Table 3, Fig. 4). Within the group of responders (n = 16), it was also found that PAI-1 levels were not significantly changed by escitalopram (*P* = 0.995).

#### Correlations of serum PAI-1 expression with clinical factors in MDD patients

The correlation analysis showed that there was no significant correlation between the serum PAI-1 levels and HDRS scores in MDD patients (r = −0.007, *P* = 0.980). Moreover, no significant correlations were found among the age, education, body weight, episodes, duration of illness and PAI-1 levels in the MDD patients (all *P* > 0.05).

## Discussion

CUMS is a valuable rodent model of depression and is widely used for antidepressant screening^22^,^23^. Our study demonstrated that CUMS decreased body weight, which was consistent with other reports^14^,^24^,^25^. CUMS-induced depression-like behaviours were restored by chronic escitalopram administration, suggesting that escitalopram has a positive effect on stressed rats. Our data showed that stress increases PAI-1 expression in area 1 of the cingulate cortex and prelimbic area of the medial prefrontal cortex as well as in cornu ammonis 3 and the dentate gyrus of the hippocampus. Chronic escitalopram treatment downregulated PAI-1 expression in these brain subregions. Stress also increased the active PAI-1 concentration in the CSF. PAI-1 in the serum was higher in stressed rats than in controls, although the difference did not reach statistical significance. Escitalopram treatment decreased the active PAI-1 concentration in the serum, but not in the CSF. The baseline serum PAI-1 concentrations in MDD patients were lower compared with controls. Escitalopram had no effect on the PAI-1 concentrations in the serum of MDD patients.

Both PAI-1 and tPA are widely expressed in the central nervous system. Recent work has discovered new functions of PAI-1 and tPA in stress reaction and depression^3^,^4^,^5^. As a tPA inhibitor, PAI-1 is upregulated in depression in animal models and clinical studies^10^,^11^,^12^. Total PAI-1 contains the following two different forms of PAI-1: an active form and a latent inactive form. Only active PAI-1 can react with tPA and form inert, covalent complexes^26^. We measured total circulating PAI-1, rather than only the active form. However, a recent investigation showed that serum total and active PAI-1 were correlated^27^. Therefore, it should be considered that the measurement of PAI-1 levels reflected the amount of active PAI-1. We found that exposure to chronic stress increased PAI-1 levels in area 1 of the cingulate cortex and the prelimbic area of the medial prefrontal cortex as well as cornu ammonis 3 and the dentate gyrus of the hippocampus. These results were consistent with a recent study that reported a rise of PAI-1 protein expression in the whole prefrontal cortex and whole hippocampus of CUMS rats^10^.

Research on animals with depression-like behaviours and MDD patients has shown that the medial prefrontal cortex network, especially the medial prefrontal cortex and hippocampus, plays a crucial role in regulating the responses to stress^28^,^29^,^30^,^31^. PAI-1 is a key inhibitor in the BDNF lysis pathway^5^,^7^. The BDNF signalling system has been associated with depression and regulates neuronal plasticity and survival. Our previous study also found that CUMS could change synaptic ultrastructural plasticity in the same brain areas of rats^14^. Therefore, the high levels of PAI-1 might inhibit the tPA/plasminogen system and inhibit cleavage of proBDNF, resulting in impaired BDNF signalling, which could result in reduced neural plasticity in the medial prefrontal cortex circuits in depression. In addition, increased PAI-1 might directly impair synaptic plasticity involved in depression. This is due to the fact that tPA also exerts various effects on synaptic plasticity through independent proteolytic activity^5^,^7^,^32^, and the PAI-1 could also interact with tPA as a binding partner to mediate these effects^5^. Moreover, depression is hypothesized to involve inflammatory processes with elevated proinflammatory cytokines, such as tumor necrosis factor-α and interleukins^33^. PAI-1 expression was also reported to be upregulated by these cytokines^34^. Therefore, the influence of PAI-1 levels by the cytokines after stress needs further study.

We also showed for the first time that chronic treatment with escitalopram can downregulate PAI-1 expression in the medial prefrontal cortex and hippocampus subregions, suggesting that PAI-1 might represent a common modulator for antidepressant action. The prefrontal cortex and the hippocampus have been postulated to be key sites where antidepressants exert their therapeutic effects^30^,^35^. Furthermore, escitalopram is reported to have anti-inflammatory activity^36^. Therefore, escitalopram might reverse the depression-like behaviour by modulating the tPA/PAI-1 system, through a mechanism that might involve the suppression of proinflammatory cytokines.

We report for the first time that active PAI-1 might undergo similar changes in the CSF and serum to the changes in brain tissues after CUMS, although the difference did not reach statistical significance. However, escitalopram reversed the level of active PAI-1 in the serum, but not in the CSF. Because CSF is produced in the choroid plexus, it can be expected that CSF PAI-1 might be secreted by neuronal cells and also by the plexus cells under the right conditions, such as escitalopram treatment. It is currently unclear if PAI-1 is capable of crossing the blood brain barrier^26^; however, PAI-1 is found in certain brain regions and in the CSF^37^,^38^. Circulating PAI-1 reflects the output of several sources, including the liver, vascular endothelium, adipose tissue and platelets^39^. Similar changes in PAI-1 levels were observed in the serum and in the brains of stressed rats, supporting its value for investigating the neurobiology and pathophysiology of MDD.

We also found that the serum of MDD patients has higher PAI-1 concentrations than that of healthy controls. Escitalopram treatment has no effect on the elevated PAI-1 concentrations. The increased PAI-1 in depressed patients was similar to the changes in the medial prefrontal cortex and hippocampus in the brains of stressed rats. This result was consistent with previous studies that demonstrated that both women^11^ and men^12^ with MDD had higher levels of PAI-1. However, subjects with higher depression scores did not have higher PAI-1 levels. Elevated PAI-1 concentrations in patients were not significantly changed with escitalopram treatment, even in the responders, which were decreased in rats’ serum by escitalopram. PAI-1 is also modulated by glucocorticoids, aldosterone and angiotensin^40^. Different levels of these factors in patients might affect the effect of escitalopram treatment on the PAI-1 concentrations. There are many other confounding factors in patients that might contribute to the variance, such as differences in episodes, duration of illness, severity of illness, drug sensitivity of different subjects and co-treatment with benzodiazepines. Comparing the life spans of the two species, longer treatment might be required to reduce the serum PAI-1 in human patients because patients received a relatively short treatment with escitalopram compared with rats in this study.

Furthermore, it has been found that PAI-1 levels are altered in disorders characterized by learning and memory deficits and by neuronal deterioration, such as Alzheimer’s disease^5^. PAI-1 levels are also increased in addiction^5^. It is possible that dysfunction of the tPA/PAI-1 system might represent a common pathophysiological mechanism shared by several disease processes.

There are some imitations to this study. First, we did not investigate other brain regions related to mood, such as the amygdala. Therefore, the changes in the medial prefrontal cortex and hippocampus might not fully represent the effects in other regions or neural circuits during a depressive episode or response to antidepressant treatment. Second, PAI-1 level is also modulated by glucocorticoids, aldosterone and angiotensin^40^. However, we did not screen for the levels of these factors in the patients and controls, which should be take into consideration in the future study. Third, all patients studied were treated with benzodiazepines, whose effect on PAI-1levels is unknown. Finally, the number of patients with MDD was small, requiring confirmation in larger cohorts.

## Additional Information

**How to cite this article**: Jiang, H. *et al*. Plasminogen Activator Inhibitor-1 in depression: Results from Animal and Clinical Studies. *Sci. Rep.* **6**, 30464; doi: 10.1038/srep30464 (2016).

## Supplementary Material

Supplementary Information

## Figures and Tables

**Table 1:** Results of behaviour tests among different groups (M ± SEM).

	Normal + saline	Normal + Esc	CUMS + saline	CUMS + Esc
Body weight (g)-0w	228 ± 6	234 ± 8	233 ± 6	221 ± 7
Body weight (g)-4w	359 ± 12	393 ± 4**	324 ± 7	326 ± 14^##^
Body weight (g)-8w	417 ± 18**	437 ± 23	355 ± 14	338 ± 17**
Sucrose preference (%)-0w	90 ± 1	93 ± 1	89 ± 1	91 ± 2
Sucrose preference (%)-4w	86 ± 2	87 ± 3	74 ± 4	65 ± 7^#△^
Sucrose preference (%)-8w	94 ± 1**	90 ± 2	49 ± 8	85 ± 3**
Immobility time (second)-0w	26 ± 4	19 ± 4	28 ± 3	15 ± 3
Immobility time (second)-4w	21 ± 3*	9 ± 2**	71 ± 14	76 ± 16^##△△^
Immobility time (second)-8w	18 ± 2**	14 ± 3	64 ± 12	26 ± 8**
Total distance (cm)-0w	1888 ± 226	2251 ± 122	2107 ± 288	2184 ± 174
Total distance (cm)-4w	1505 ± 194**	1153 ± 216**	465 ± 93	424 ± 52^##△△^
Total distance (cm)-8w	1373 ± 129**	1575 ± 193	309 ± 103	1190 ± 125**
Rearing number-0w	27 ± 4	29 ± 3	27 ± 3	22.50 ± 3.01
Rearing number-4w	18 ± 3**	22 ± 3*	2 ± 1	5 ± 1^△##^
Rearing number-8w	18 ± 2**	14 ± 2	5 ± 2	12 ± 1*

Notes: M: mean; SEM: standard error of mean; Esc: escitalopram; CUMS: chronic unpredictable mild stress. Compared with “CUMS + saline” group, *P < 0.05, **P < 0.01. Compared with “Normal + saline” group, ^#^*P* < 0.05, ^##^*P* < 0.01. Compared with “Normal ^+^ Esc” group, ^△^*P* < 0.05, ^△△^*P* < 0.01.

**Table 2:** PAI-1 levels in several brain subregions, cerebrospinal fluid and serum of rats among different groups (M ± SEM).

	Normal + saline	Normal + Esc	CUMS + saline	CUMS + Esc
Cg1	0.22 ± 0.01	0.10 ± 0.00	0.88 ± 0.02**	0.67 ± 0.02^##^
PrL	0.32 ± 0.02	0.35 ± 0.02	0.81 ± 0.04**	0.55 ± 0.03^##^
CA1	0.43 ± 0.02	0.57 ± 0.02	0.25 ± 0.01**	0.27 ± 0.01
CA3	0.26 ± 0.01	0.12 ± 0.00	1.00 ± 0.04**	0.47 ± 0.02^##^
DG	0.60 ± 0.02	0.40 ± 0.01	0.93 ± 0.04**	0.69 ± 0.02^##^
CSF (ng/ml)	1.38 ± 0.22	2.95 ± 0.51	3.55 ± 0.32**	3.44 ± 0.18
Serum (ng/ml)	1.16 ± 0.17	4.66 ± 0.48	2.50 ± 0.50	0.74 ± 0.10^#^

Note: The protein levels of PAI-1 in Cg1, PrL, CA1, CA3 and DG were normalized to GAPDH. M: mean; SEM: standard error of mean; Esc: escitalopram; CUMS: chronic unpredictable mild stress; Cg1: area 1 of the cingulate cortex; PrL: prelimbic cortex; CA1: cornu ammonis 1; CA3: cornu ammonis 3; DG: dentate gyrus. CSF: cerebrospinal fluid.

Compared with “Normal + saline” group by post-hoc analysis, *P <0.05, **P < 0.01. Compared with “CUMS + saline” group by post-hoc analysis, ^#^*P* < 0.05, ^##^*P* < 0.01.

**Table 3:** Characteristics of MDD patients and controls (M ± SD).

	Controls (n = 17)	MDD (n = 17)	
		Before treatment	After treatment
Age (years)	54 ± 3	48 ± 12^a^	—
Gender (males/females)	4/13	3/14^b^	—
Education (years)	10 ± 2	13 ± 4^a^	—
Body weight (kg)	67 ± 7	62 ± 15^a^	—
Duration of illness (months)	—	44.5 ± 70.0	—
Episodes	—	1.5 ± 0.9	—
HDRS	2.4 ± 1.8	21.6 ± 6.3**^a^	4.5 ± 6.0^##^^c^
PAI-1 (pg/ml)	4.5 ± 1.6	6.2 ± 2.0*^a^	6.0 ± 2.2^c^

Notes: M: mean; SD: standard deviation; MDD: major depressive disorders; HDRS: 17-item Hamilton Depression Rating Scale.

Compared with controls, *P < 0.05, **P < 0.01. Compared with “before treatment”, ^#^*P* < 0.05, ^##^*P* < 0.01.

^a^Analysis of variance.

^b^Chi-square test.

^c^Paired-samples t-test.
