# Supplementary material for: Plasminogen Activator Inhibitor-1 in depression: Results from Animal and Clinical Studies
Source: Sci Rep. 2016 Jul 26;6:30464. doi: 10.1038/srep30464 (PMC4960524; doi:10.1038/srep30464)
Supplement: Supplementary Information [file srep30464-s1.doc]

**Plasminogen Activator Inhibitor-1 in depression: Results from Animal and Clinical Studies**

Haitang Jiang, MD^1,2^, Xiaoli Li, MD, PhD^3^, Suzhen Chen, MD^1,2^, Na Lu, MD^1,2^, Yingying Yue, MD^1,2^, Jinfeng Liang, MD^1,2^, Zhijun Zhang, MD, PhD ^3^*, Yonggui Yuan, MD, PhD^1,2^*

**Table S1.** **The results of two-way ANOVA of PAI-1 levels in several brain subregions, cerebrospinal fluid and serum of rats in four groups (M±SEM).**

|  | Stress factor | |  | Drug factor | |  | Main effect | | Interactive  effect  *P* value |
| --- | --- | --- | --- | --- | --- | --- | --- | --- | --- |
|  | Absence  (n=10) | Presence  (n=10) |  | Absence  (n=10) | Presence  (n=10) |  | Stress  *P* value | Drug  *P* value |  |
| CG1 | 0.16 ± 0.02 | 0.77 ± 0.04 |  | 0.55 ± 0.11 | 0.38 ± 0.10 |  | <0.001 | <0.001 | 0.004 |
| PrL | 0.34 ± 0.01 | 0.68 ± 0.05 |  | 0.56 ± 0.08 | 0.45 ± 0.04 |  | <0.001 | <0.001 | <0.001 |
| CA1 | 0.50 ± 0.03 | 0.26 ± 0.01 |  | 0.34 ± 0.03 | 0.42 ± 0.05 |  | <0.001 | <0.001 | 0.003 |
| CA3 | 0.19 ± 0.02 | 0.73 ± 0.09 |  | 0.63 ± 0.12 | 0.29 ± 0.06 |  | <0.001 | <0.001 | <0.001 |
| DG | 0.50 ± 0.04 | 0.81 ± 0.05 |  | 0.76 ± 0.06 | 0.55 ± 0.05 |  | <0.001 | <0.001 | 0.401 |
| CSF (ng/ml) | 2.17 ± 0.36 | 3.49 ± 0.18 |  | 2.46 ± 0.38 | 3.20 ± 0.27 |  | 0.039 | <0.001 | 0.020 |
| Serum (ng/ml) | 2.91 ± 0.58 | 1.62 ± 0.36 |  | 1.83 ± 0.32 | 2.70 ± 0.64 |  | 0.025 | 0.002 | <0.001 |

ANOVA: Analysis of variance; M: mean; SEM: standard error of mean; CG1: area 1 of the cingulate cortex; PrL: prelimbic cortex; CA1: Cornu Ammonis 1; CA3: Cornu Ammonis 3; DG: dentate gyrus. CSF: cerebrospinal fluid.
